# Inter-kingdom interactions and environmental influences on the oral microbiome in severe early childhood caries

**DOI:** 10.1128/spectrum.02518-24

**Published:** 2025-04-15

**Authors:** Lingjia Weng, Yuqi Cui, Wenting Jian, Yuwen Zhang, Liangyue Pang, Yina Cao, Yan Zhou, Wei Liu, Huancai Lin, Ye Tao

**Affiliations:** 1 Hospital of Stomatology, SunYat-sen University 623167 , Guangzhou, Guangdong, China; 2 Guangdong Provincial Key Laboratory of Stomatology SunYat-sen University 540446 , Guangzhou, Guangdong, China; 3 Guanghua School of Stomatology, Sun Yat-Sen University 540445 , Guangzhou, Guangdong, China; 4 Department of Cardiovascular Surgery, Sun Yat-sen Memorial Hospital, Sun Yat-sen University 56713 , Guangzhou, Guangdong, China; University of Florida College of Dentistry, Gainesville, Florida, USA

**Keywords:** early childhood caries, microbial ecology, bacterial-fungal interactions, fungi, bacteria, saliva, dental plaque, *Candida* species

## Abstract

Dental caries arise from intricate interactions among oral microorganisms, impacting ecological stability and disease progression. In this study, we aimed to investigate the microbial diversity and inter-kingdom interactions in severe early childhood caries (S-ECC) and assess the influence of environmental factors such as salivary pH and trace elements. We analyzed 61 children aged 3–4 years with complete deciduous dentition, evaluating salivary pH, buffering capacity, and trace elements (iron, fluoride). We examined the performance of 16S rRNA V1–V9 regions gene and internal transcribed spacer (ITS) primers for bacteria and fungi from plaque and saliva to characterize community compositions and diversity. Findings revealed significant shifts in bacterial diversity in S-ECC saliva samples, marked by decreased diversity and elevated abundance of cariogenic species, particularly *Streptococcus mutans. Candida albicans* was notably more prevalent in the S-ECC group, implicating its potential role in pathogenesis. Iron and fluoride concentrations showed no significant correlation with microbial community structure. Network analyses uncovered complex intra- and inter-kingdom interactions, underscoring cooperative and competitive dynamics. S-ECC children exhibited higher abundances of bacteria (*Streptococcus mutans*, *Granulicatella*, *Actinomyces*) and fungi (*Candida albicans*), with specific microbial taxa associated with reduced salivary pH.

**IMPORTANCE:**

This study illuminates the intricate relationship between bacteria and fungi within the oral microbial community of children, specifically highlighting differences between those with S-ECC and those without caries. Through an extensive analysis of the microbial composition in both saliva and dental plaque, we identified a significant increase in the abundance of specific bacterial taxa (e.g., *S. mutans, Granulicatella, Actinomyces*) and fungal species (e.g., *C. albicans*) in the oral cavities of children with S-ECC. This finding underscores the potential role of these microorganisms in the development of caries. Contrary to previous studies that emphasize the importance of iron and fluoride in oral health, our research found no significant correlation between the concentrations of these elements and the composition of oral microbial communities. This result challenges conventional understanding and opens new avenues for future research. Additionally, our findings revealed an association between *Veillonella* sp., *Propionibacterium* sp., and *Candida* sp. and reduced salivary pH. This provides novel insights into the relationship between the oral microenvironment and caries development. The implications of our findings are substantial for the development of prevention and intervention strategies targeting childhood caries. They also underscore the critical need for a deeper exploration of oral microbial interactions and their environmental influences.

## INTRODUCTION

Dental caries, a prevalent oral disease, is particularly concerning in the context of severe early childhood caries (S-ECC), which affects children with complete deciduous dentition. The ecological plaque hypothesis asserts that dental caries originate from a symbiotic yet dynamic consortium of microorganisms, including but not limited to fungi and bacteria, within the oral cavity (1, 2). These microorganisms contribute to oral ecological stability or disease progression via symbiotic relationships, competitive dynamics, and antagonistic interactions. Our previous exploratory study revealed differences in fungi from different ecological sites (3), highlighting the need for a comprehensive analysis of the saliva and dental plaque microbiota in caries-free and S-ECC children.

Recent research highlighted the intimate interactions between fungi and bacteria, which coalesce into robust three-dimensional biofilms, thereby augmenting their pathogenicity (4–6). The fungal-bacterial nexus has recently piqued interest in the pathogenetic landscape of oral infectious diseases (7–9). Notably, *Enterococcus faecalis*, frequently isolated alongside *Candida albicans* (*C. albicans*) in the oral cavity has been shown to adhere to *C. albicans* hyphae, with *in vitro* studies demonstrating enhanced biofilm formation (10–12) and tolerance to alkaline environments, potentially exacerbating periapical inflammatory responses (13, 14). *Streptococcus mutans* (*S. mutans*), a notorious cariogenic bacterium, has been implicated in a potential synergistic relationship with *C. albicans*, particularly in the oral cavity of children with S-ECC (15, 16). However, reports also indicate an antagonistic relationship between the two, where compounds secreted by *S. mutans* may inhibit *C. albicans* growth (17–19). Additionally, other *Streptococcus* species such as *Streptococcus sanguinis*, *Streptococcus gordonii*, and *Streptococcus oralis* have also been reported to synergistically enhance *C. albicans* overgrowth (20–23), although the underlying mechanisms remain to be elucidated. While numerous studies have been conducted *in vitro*, the intricate interplay between oral bacterial and fungal communities is not fully understood. High-throughput sequencing technologies now provide tools for dissecting these interactions at the omics level, providing a holistic view of the microecological etiology of caries.

Saliva is widely used for caries sampling studies (24). In addition to the flora information contained in saliva, salivary pH, buffering capacity, and trace elements are critical to maintaining the stability of the oral ecosystem. The salivary buffering system, exemplified by bicarbonate, ensures oral pH homeostasis (25). Excessive sugar intake can lead to a decrease in pH due to carbohydrate fermentation by acidogenic bacteria, resulting in tooth surface demineralization and caries development (26, 27). Research indicates that pH reduction can alter bacterial community structures and decrease diversity, with an increase in *Firmicutes* and *Lactobacillus* under acidic conditions (28, 29). A study suggested that a core model comprised of approximately 60% of the oral taxa contributes to the deleterious shift in bacterial diversity to acidic (cariogenic) from neutral (healthy) pH environments (30). This shift is associated with an increase in acid-producing bacterial species, such as *Veillonella*, *Lactobacillus*, and *Propionibacterium*, which become part of the dental biofilm and contribute to cariogenic conditions in the mouth by producing acids (31).

Previous studies have identified iron as a crucial nutrient for bacterial and fungal growth (32, 33), with many pathogenic bacteria possessing efficient iron uptake systems that may concurrently disadvantage beneficial bacteria (34, 35). Iron supplementation has been suggested as a means to thwart the progression of caries (36, 37). The study proved that iron availability can modulate host-associated oral microbial communities, resulting in a microbiota with potential clinical impact (38). Similarly, fluoride is recognized for its role in caries prevention, primarily by hindering enamel demineralization and promoting remineralization (39–41). However, the impact of fluoride on complex *in vivo* biofilm communities may be less evident than previously thought.

Given the multifactorial nature of dental caries and the complex interplay between oral microbiota, environmental factors, and host responses, a comprehensive understanding of these interactions is essential for developing effective preventive strategies. Here, we comprehensively analyzed the composition of the saliva and dental plaque microbiota of caries-free and S-ECC children aged between 3 and 4 years. This study aims to (i) compare the differences in bacterial and fungal diversity between different ecological sites in the S-ECC and caries-free groups, (ii) understand the intricate relationships within oral bacterial and fungal communities, and (iii) investigate the interplay between salivary pH, iron, fluoride, and oral microbial communities. By addressing these objectives, we hope to provide insights into the microecological etiology of caries and identify potential targets for therapeutic interventions.

## MATERIALS AND METHODS

### Population

Sixty-one children aged 3 to 4 years from two kindergartens in Zengcheng District, Guangzhou, with complete deciduous dentition were recruited for this study. The exclusion criteria were as follows: (i) children with systemic disease or those who had used antibiotics within 3 months; (ii) children with salivary gland disease and/or other oral diseases (such as periodontitis [42] and oral mucosal disease); (iii) children who applied topical and systemic fluoride within 6 months; and (iv) children with primary teeth with enamel hypoplasia (43). Children’s general oral hygiene habits and dietary habits were investigated through a structured questionnaire (Supplement 1) administered to their guardians or caregivers. Written informed consent was obtained from the guardians of all participants, and ethical approval for the study was obtained from the Ethics Committee of the Hospital of Stomatology, Sun Yat-sen University, in Guangzhou, China (KQEC-2020-60-02).

### Clinical examination

The International Caries Detection and Assessment System II criteria were adopted for clinical examination (3). Twenty-eight children with S-ECC (decayed, missing, or filled tooth surfaces [dmfs] ≥ 4) and 33 caries-free (dmfs = 0) children participated in the study (44). Two experienced dentists (Cui Y. and Zhang Y., kappa: 0.80–0.89) performed oral examinations using a standard mouth mirror, headlamp, and community periodontal index probe. The visible plaque index was used to examine all the teeth of the sample population, including the distal, medial, proximal, and lingual or palatal surfaces of each tooth under a natural light source. The results were expressed as a percentage of the total number of examined sites with visible plaque.

### Sample collection

**Fig 1:**
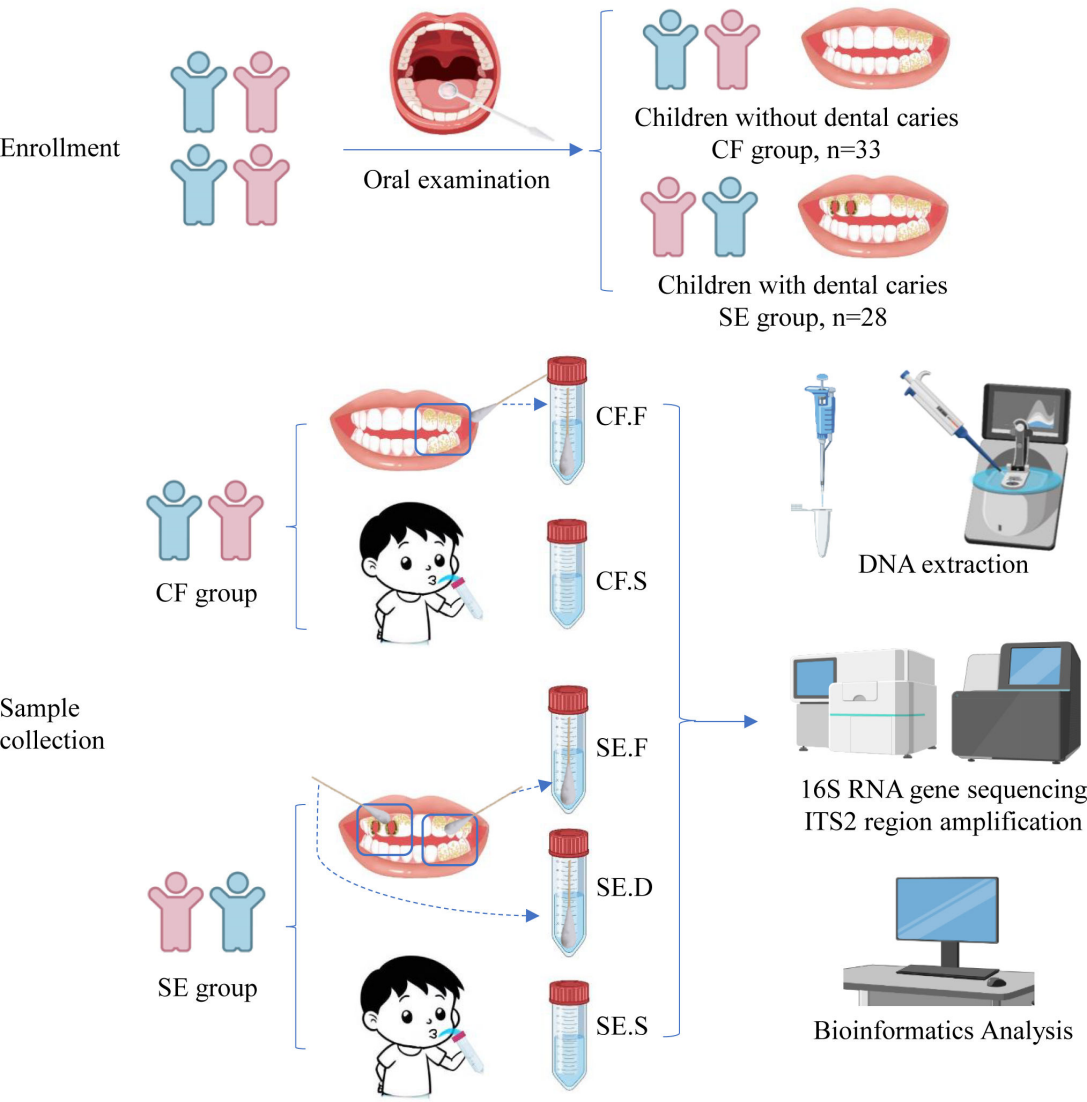
Eligible children were enrolled in the study and divided into the severe early (SE) and caries-free (CF) groups after oral examination. In both groups, mixed supragingival plaque and nonirritating saliva were collected from available healthy tooth surfaces. In the SE group, mixed supragingival plaque from caries-damaged tooth surfaces was also collected. The samples from different groups and sections were then subjected to DNA extraction and analysis.

All samples were collected between 8 a.m. and 9 a.m. (Fig. 1). Children were required to refrain from tooth brushing for 12 ± 4 h and avoid eating or drinking for 2 h before sampling.

The pH and buffering capacity of saliva were measured as follows: a caries indicator test strip (Xierou, China) was placed in the child’s mouth, and the test strip was wetted with saliva. After the color of the test strip stabilized (usually 10 s), the test strip was compared with the standard colorimetric card on the packaging bottle, and the values were recorded.

Samples of supragingival plaque were collected from tooth surfaces and categorized as follows: mixed supragingival plaque from available healthy tooth surfaces in both the caries-free (CF) and severe early (SE) groups (F), and mixed plaque from decayed tooth surfaces in the SE group (D), were collected using a sterile cotton swab. All plaque samples were then placed in a DNA-free TE buffer (pH = 7.4). After all plaque samples were collected, 5 mL of saliva (S) was collected using 15 mL sterile centrifuge tubes from all individuals.

All biological samples were immediately placed on dry ice and transferred to a −80°C freezer for storage before further analysis. The samples collected represent five different spatial niches: three in the SE group (SE.F, *n* = 28; SE.D, *n* = 28; SE.S, *n* = 28) and two in the CF group (CF.F, *n* = 33; CF.S, *n* = 33).

### Ion concentration measurement in saliva

#### Fluorine ion concentration measurement

The instrument was calibrated before the assay. The gradient dilution of the NaF solution was used to calibrate the fluoride ion concentration, which was calculated as *y = 58x*−*12*. Saliva samples at −80°C were thawed at room temperature and then diluted for the assay.

#### Iron ion concentration measurement

The standard curve of the Fe concentration measured with gradient-diluted FeNO_3_ solution using an inductively coupled plasma mass spectrometer (ICP-MS) yielded a correlation coefficient R^2^ of 0.997 and the regression equation of *y = 752.184x* + *49.607* for Fe. The iron concentration in saliva was determined using ICP-MS.

### Laboratory methods

DNA was extracted using a DNA extraction kit (Takara Bio, China) according to the protocol of the manufacturer. The DNA concentration and purity were measured using the NanoDrop One (Thermo Fisher Scientific, USA).

For bacterial community analysis, a portion of the 16S rRNA V1–V9 regions was amplified with the barcoded universal primers (27F-AGRGTTYGATYMTGGCTCAG and 1492R-RGYTACCTTGTTACGACTT). The fungal internal transcribed spacer (ITS2) region was amplified using PCR. The forward primer sequence (gITS7ngs-GTGARTCATCRARTYTTTG) and the reverse primer sequence (ITS4ngs-TCCTSCGCTTATTGATATGC) were shown in our previous study (3, 45). PCR reactions, containing 25 µL 2× Premix Taq (Takara Bio, China), 1 µL each primer (10 µM) and 3 µL DNA (20 ng/µL) template in a volume of 50 µL, were amplified by thermocycling: 5 min at 94°C for initialization; 30 cycles of 30 s denaturation at 94°C, 30 s annealing at 52°C, and 30 s extension at 72°C; followed by 10 min final elongation at 72°C. The PCR instrument was a BioRad S1000 (Bio-Rad Laboratory, USA).

The length and concentration of the PCR products were detected by 1% agarose gel electrophoresis. Samples with bright main strips were selected for further experiments, while those without bright bands were removed. PCR products were mixed in equidensity ratios according to the GeneTools Analysis Software (Version 4.03.05.0, SynGene). Then, the mixture of PCR products was purified with the E.Z.N.A. Gel Extraction Kit (Omega, USA).

According to the 16S Amplification SMR Tbell Library Preparation and NEBNext Ultra II DNA Library Prep Kit for Illumina (New England Biolabs, USA) standard process database building operations, sequencing of the constructed amplicon library using the PacBio Sequel II and Illumina Nova 6000 Nova platforms (Magigene Biotechnology Co., China).

### Bioinformatics analysis

#### Sequencing data processing

The V1–V9 amplified sequences were split, corrected, formatted, converted, and removal of host sequences and sequences that did not match the upper primers to obtain the final valid data by using PacBio’s SMRT Link (Version 6.0). Fastp (Version 0.14.1, https://github.com/OpenGene/fastp) was used to cut the raw data from the ITS2 region amplification to obtain the valid splice fragment.

#### OTU cluster and species annotation

The valid data of all samples were clustered with 97% agreement using USEARCH software (V10, http://www.drive5.com/usearch/) and annotated with the HOMD database (http://www.homd.org). Details of the analysis of ITS2 region amplification data were shown in our former study (3).

#### Alpha and beta diversity

The alpha and beta diversity were analyzed using QIIME software (Version 1.9.1). Statistical differences in alpha and beta diversity were analyzed using Wilcoxon rank-sum tests between categories by pairwise comparison. Principle coordinates analysis (PCoA) and nonmetric multi-dimensional scaling (NMDS) diagrams were drawn using R software (Version 2.15.3). Significant differences in species abundance between categories were analyzed by the linear discriminant analysis effect size (LEfSe) method; results were statistically significant when *P* < 0.05.

#### Environmental factors correlation analysis

Iron concentration and fluoride concentration were used as quantitative variables. Redundancy analysis (RDA) and Mantel test analysis were used to investigate their correlation and influence on the distribution of oral microbial communities.

Salivary pH and buffering capacity were categorical variables. Multivariate association with linear models (MaAsLin) analysis (46, 47) was used to plot the correlation heatmap with a relative abundance threshold set at 0.01%. MaAsLin analysis is an analytical method used to effectively determine multivariate associations between clinical data and microbiome characteristics. All *P* values were corrected for multiple comparisons using false discovery rate (FDR).

#### Network prediction analysis

Based on the abundance of species in each group of samples, microbial species with relative abundances greater than 0.01% were selected. Then, R software was used to calculate the Spearman correlation coefficient to determine the correlation between species within the sample group. Bacterial and fungal species with |SpearmanCoef| > 0.6 and *P* values less than 0.01 were subjected to covariance network analysis.

### Questionnaire analysis

The questionnaire data were entered into Excel and analyzed with SPSS software (Version 25.0). The data were expressed as the mean ± standard deviation. Differences were considered statistically significant when the two-sided *P* value was less than 0.05 for all statistical analyses.

## RESULTS

### Overview of the subjects and samples

A total of 61 children aged 3–4 years were included in this study, with a mean age of 44.4 ± 4.7 months, including 33 in the CF group and 28 in the SE group. The dmfs in the SE group ranged from 4 to 21, and the mean ± SD was 9.36 ± 4.80. There were no significant differences in oral hygiene habits, oral hygiene status, or dietary habits between the two groups. Statistically, a higher proportion of children in the SE group were born by caesarean section and breastfed within 6 months after birth (*P* < 0.05) (Table 1).

**TABLE 1:** Comparison of socio-economic background and behavioral differences between the two groups^*d*^^,*e*^

Variable	CF group (*n* = 33)	SE group (*n* = 28)
Age (month)^*a*^	43.55 ± 4.80	45.5 ± 4.39
Weight when born^*b*^		
<1.5 kg	0	1 (3.6)
1.5–2.5 kg	5 (15.2)	3 (10.7)
>2.5 kg	28 (84.8)	24 (85.7)
Gestational age^*c*^		
28–36 weeks	5 (15.2)	6 (21.4)
>37 weeks	28 (84.8)	22 (78.6)
Mode of delivery^*c*^		
Cesarean section	13 (39.4)	21 (75.0)**
Natural birth	20 (60.6)	7 (25.0)
Feeding patterns within 6 months^*b*^		
Breastfeeding	13 (39.4)	15 (53.6)^*^
Mixed feeding	18 (54.5)	7 (25.0)
Pure milk powder feeding	2 (6.1)	6 (21.4)
Abstain from night feedings^*c*^		
≤2 years	23 (69.7)	20 (71.4)
>2 years	10 (30.3)	8 (28.6)
Toothbrushing frequency^*c*^		
≤1 time/day	23 (69.7)	14 (50.0)
≥2 times/day	10 (30.3)	14 (50.0)
Frequency of intake of sweets^*c*^		
1–3 times/week	24 (72.7)	22 (78.6)
>3 times/week	9 (27.3)	6 (21.4)
Monthly income per household^*c*^		
<5,000 yuan	6 (18.2)	6 (21.4)
≥5,000 yuan	27 (81.8)	22 (78.6)
Visual dental plaque^*b*^		
≥20%	31 (94.0)	27 (96.4)
<20%	0 (0)	1 (3.6)
0%	2 (6.0)	0 (0)

^*a*^ *t*-test.

^*b*^ Fisher’s test.

^*c*^ Chi-square test.

^*d*^ *P < 0.05, **P < 0.01.

^*e*^ Values are mean ± SD or *n* (%).

### Sequencing information

A total of 285 samples (136 for bacteria and 149 for fungi) were selected for analyses. A total of 844,232 valid sequences were generated by PacBio Sequel II sequencing, with an average of 6,208 sequences per sample. Among all the samples, the maximum number of sequences was 12,021, while the minimum number was 2,603. A total of 4,086,821 effective sequences were generated by Illumina Nova600 sequencing, with an average of 27,428 sequences per sample. Among all the samples, 74,620 sequences were the most, and 1,271 sequences were the least.

### Composition of oral bacterial and fungal communities at different spatial positions in the SE and CF groups

**Fig 2:**
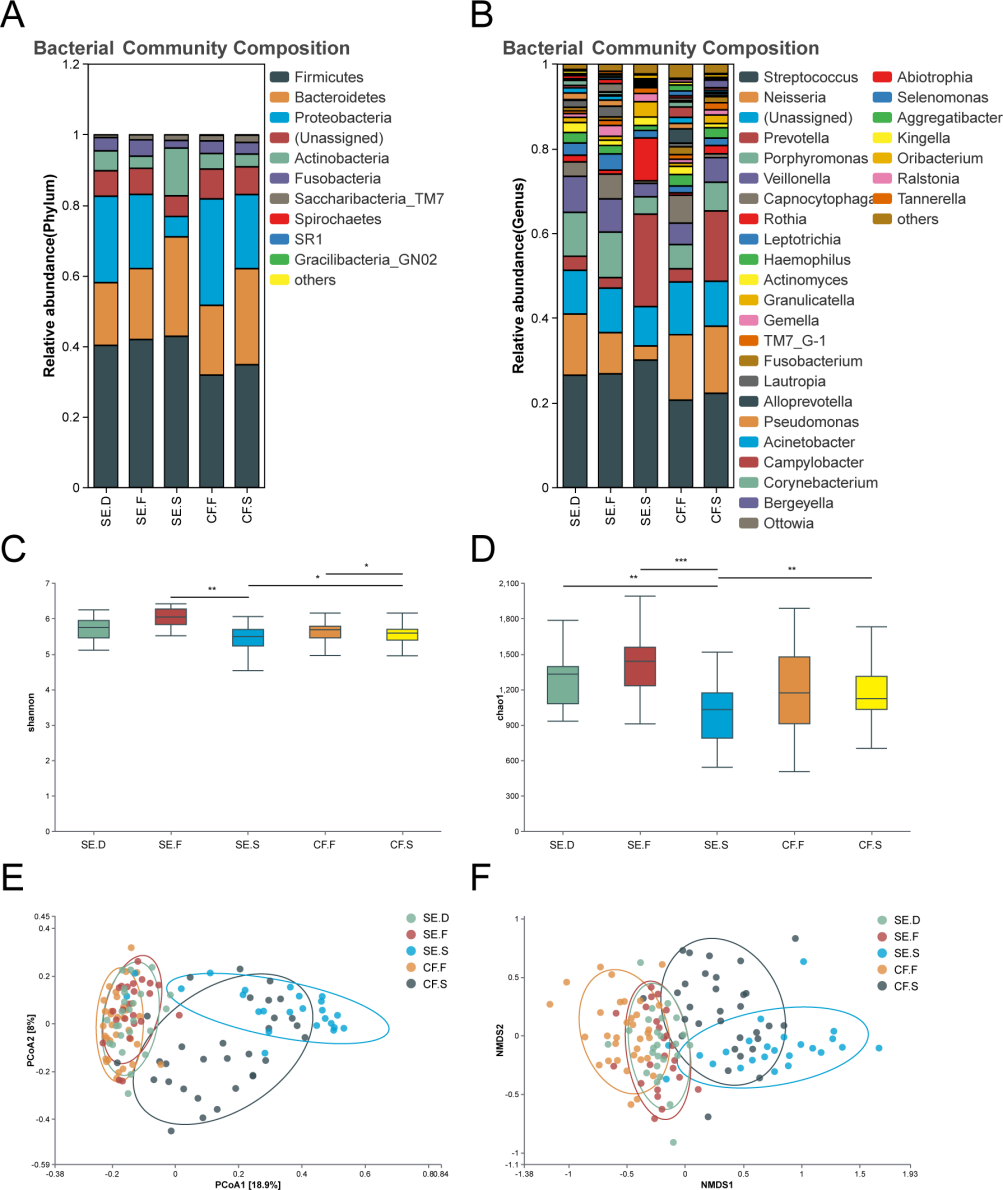
Species diversity of bacteria in various spatial locations of the oral cavity. Bacterial phylum (**A**) and genus (**B**) composition of the SE.C, SE.F, SE.S, CF.F, and CF.S groups. The Alpha diversity index of each group was compared using the Shannon (**C**) and Chao1 indices (**D**). The distribution of the oral bacterial community in each group is shown through bacterial community PCoA (**E**) and NMDS analysis (**F**). The analysis revealed that the bacterial community structures of saliva and plaque samples were completely separate from each other.

Bacteria: a total of nine phyla were found in the overall bacterial community of all plaque and saliva samples—Firmicutes, Bacteroidetes, Proteobacteria, Actinobacteria, Fusobacteria, Saccharibacteria_TM7, Spirochaetes, Absconditabacteria_(SR1), and Gracilibacteria_(GN02). Among the five subgroups of the CF and SE groups, Firmicutes had the highest abundance (CF.S: 34.8%, CF.F: 31.9%, SE.S: 42.9%, SE.F: 42.0%, SE.D: 40.3%). There was little difference in the abundance of each phylum among the five spatial locations (Fig. 2A).

A total of 15 dominant genera were identified in the overall microbial community of all plaque and saliva samples: *Streptococcus*, *Neisseria*, *Prevotella*, *Porphyromonas*, *Veillonella*, *Capnocytophaga*, *Rothia*, *Leptotrichia*, *Haemophilus*, *Actinomyces*, *Granulicatella*, *Gemella*, *Saccharibacteria_TM7_G-1*, *Fusobacterium*, and *Lautropia* (Fig. 2B).

**Fig 3:**
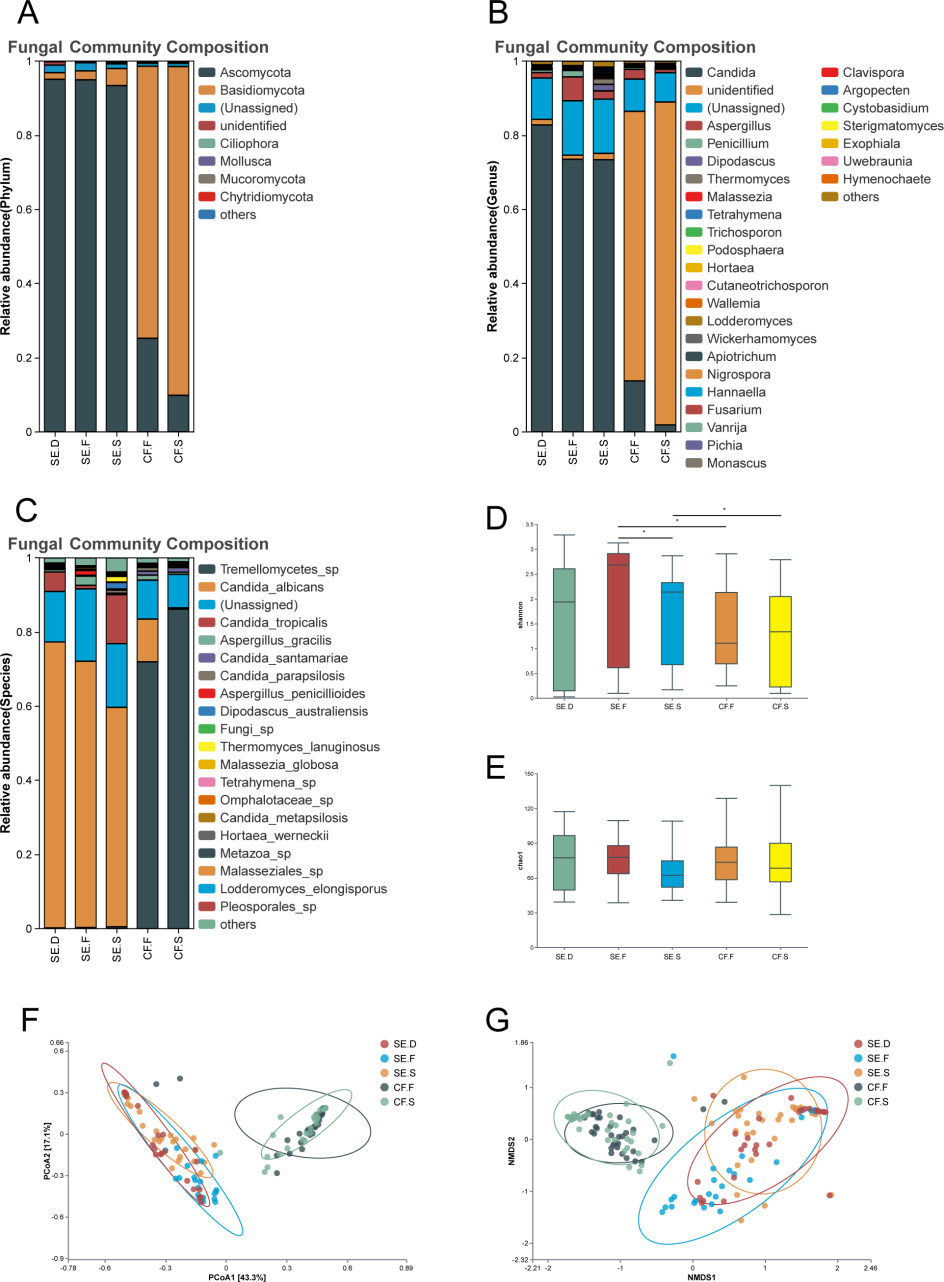
Species diversity of fungi in various spatial locations of the oral cavity. The fungal phyla (**A**), genera (**B**), and species (**C**) of the SE.C, SE.F, SE.S, CF.F, and CF.S groups were examined. The analysis compared the Shannon index (**D**) and Chao1 index (**E**) among different groups. PCoA (**F**) and NMDS analysis (**G**) were conducted to investigate the distribution status of the oral fungal communities in each group. The results showed that saliva and plaque samples have distinct fungal community structures.

Fungi: two dominant phyla were found in the overall microbial community of all plaque and saliva samples—Ascomycota (CF.S: 9.8%, CF.F: 25.2%, SE.S: 93.4%, SE.F: 95.0%, SE.D: 95.1%) and Basidiomycota (CF.S: 88.7%; CF.F: 73.4%; SE.S: 4.6%, SE.F: 2.4%; SE.D: 1.8%). The abundance of the two phyla differed significantly between the SE and CF groups. The abundance of Ascomycota was higher in the spatial positions of the SE group, while the abundance of Basidiomycota was higher in the spatial positions of the CF group (Fig. 3A). Two dominant genera (relative abundance >1%) were found in the overall microbial community of all plaque and saliva samples: *Candida* (CF.S: 1.9%, CF.F: 13.7%, SE.S: 73.4%, SE.F: 73.5%, SE.D: 82.8%) and *Aspergillus* (CF.S: 0.9%, CF.F: 2.6%, SE.S: 2.2%, SE.F: 6.4%, SE.D: 1.5%) (Fig. 3B). A total of three dominant species were found in all plaque and saliva samples: *C. albicans* (CF.S: 0.3%, CF.F: 11.5%, SE.S: 59.2%, SE.F: 71.8%, SE.D: 77.1%), Tremellomycetes sp. (CF.S: 86.2%, CF.F: 72.0%, SE.S: 0.5%, SE.F: 0.3%, SE.D. 0.2%), and *Candida tropicalis* (CF.S: 0.001%, CF.F: 0.02%, SE.F: 2.5%, SE.D: 5.2%) (Fig. 3C).

### Alpha diversity analysis

Alpha (α) diversity indicators, Chao1, and Shannon were used to assess whether overall bacterial diversity differed between samples. Our statistical analyses revealed significant differences in oral bacterial community diversity among the SE.F–SE.S, SE.D–SE.S, SE.S–CF.S, and CF.F–CF.S groups (*P* < 0.05), as illustrated in Fig. 2C and D. These findings indicate a reduced bacterial diversity in saliva samples within the SE group compared to both healthy and carious plaque samples. Additionally, saliva samples from the CF group exhibited lower bacterial diversity than healthy plaque samples. Furthermore, within the SE group, bacterial diversity in saliva samples was less than that observed in the CF group.

Similarly, the Shannon and Chao1 indices revealed significant disparities in oral fungal community diversity for SE.F–SE.S, SE.F–CF.F, and SE.S–CF.S group comparisons (*P* < 0.05), as depicted in Fig. 3D and E. These results suggest that saliva samples from the SE group possessed diminished fungal diversity when compared with healthy dental plaque samples. Conversely, the fungal diversity within healthy dental plaque samples from the CF group was lower than that in the SE group. Notably, saliva samples from the SE group exhibited higher fungal diversity than those from the CF group.

### Comparative analysis of the composition of oral bacterial and fungal communities at each spatial site in the SE and CF groups

Based on the NMDS and PCoA analysis using the Bray-Curtis distance, we described the distribution of bacterial and fungal communities in the SE and CF groups. The results of the bacteria analysis are shown in Fig. 2E and F, and Table 2. Saliva and plaque samples were completely separated in terms of bacterial community structure. The bacterial communities in saliva samples from the SE and CF groups were significantly different, as were those in the plaque samples. Multi-response permutation procedure (MRPP) statistical analysis revealed the statistically significant differences between the mentioned groups (*P <* 0.05).

**TABLE 2:** MRPP analysis of differences between groups—bacteria^*a*^

Groups	A value	*P*
SE.D–SE.F	0.005	0.066
SE.D–SE.S	0.109	<0.001
SE.F–SE.S	0.099	<0.001
SE.F–CF.F	0.019	<0.001
SE.S–CF.S	0.043	<0.001
CF.F–CF.S	0.064	<0.001

^*a*^ The differences among the groups mentioned above are larger than the differences within each group.

The results for the fungal communities are presented in Fig. 3F and G and Table 3. Saliva and plaque samples were completely separated in terms of fungal community structure. The fungal communities in saliva samples from the SE and CF groups were significantly different, as well as the plaque samples. MRPP statistical analysis showed the statistically significant differences between the mentioned groups (*P <* 0.05).

**TABLE 3:** MRPP analysis of differences between groups—fungi^*a*^

Groups	A value	*P*
SE.D–SE.F	0.016	0.036
SE.D–SE.S	0.030	0.008
SE.F–SE.S	0.033	0.005
SE.F–CF.F	0.105	<0.001
SE.S–CF.S	0.139	<0.001
CF.F–CF.S	0.014	0.061

^*a*^ The differences among the groups mentioned above are larger than the differences within each group.

### Analysis of differences in oral bacterial and fungal species by spatial site in the SE and CF groups

#### Bacteria

**Fig 4:**
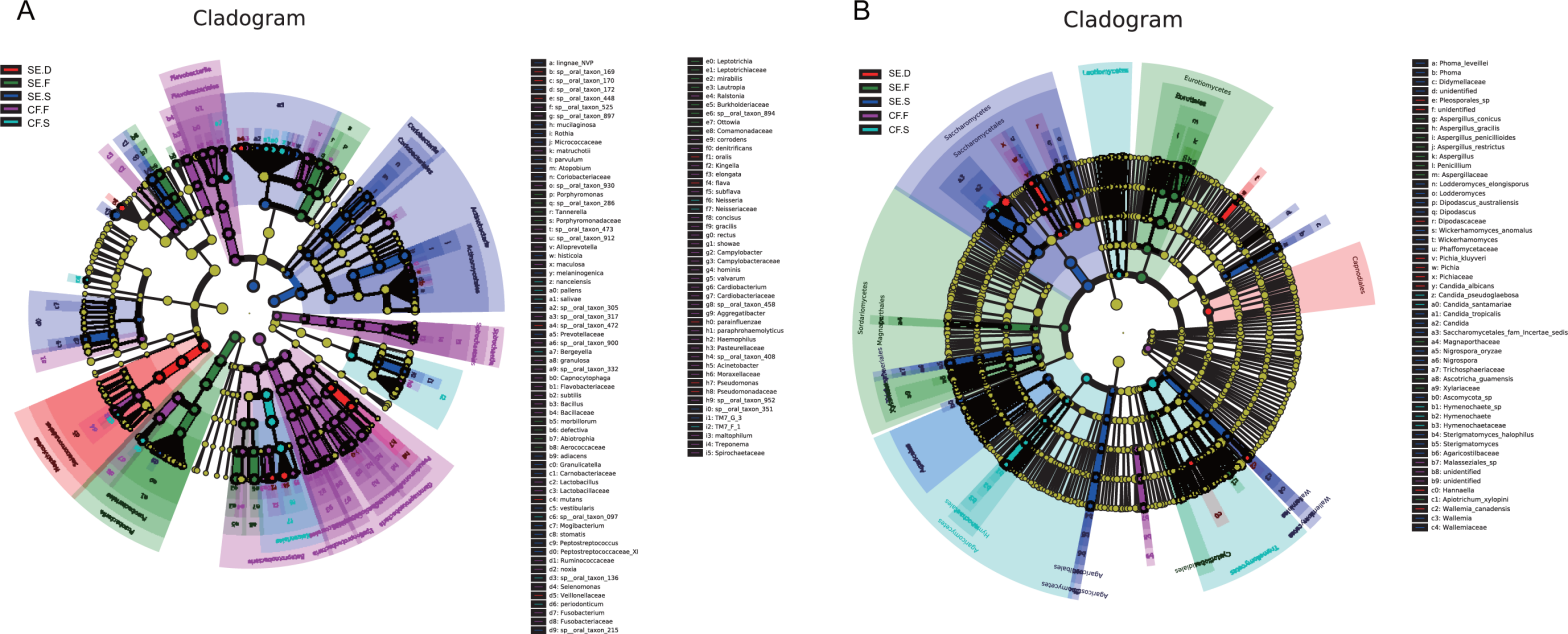
Differential bacteria (**A**) and fungi (**B**) at various sites in the oral cavities. LEfSe was used to analyze the species that differed among the groups. In the evolutionary branching diagram, circles radiating from inside to outside represent taxonomic levels from phylum to genus (or species). Each small circle at a different taxonomic level represents a taxon at that level. The size of the circle diameter is proportional to the relative abundance. Species without significant differences are uniformly colored in yellow. Different species follow the group for coloring. Different nodes are colored to represent microbial taxa that play an important role in the group. The Linear Discriminant Analysis (LDA) value is set to 3.

As shown in Fig. 4A, samples from the CF.S group accounted for a higher proportion of *Prevotella nanceiensis*, *Fusobacterium periodonticum*, *Selenomonas* sp. oral taxon 136, *Prevotella salivae*, *Neisseria* sp., *Stomatobaculum* sp. oral taxon 097, *Prevotella pallens*, and *Bergeyella* sp. than others. As for samples from the CF.F group, a higher proportion of *Selenomonas* sp., *Acinetobacter* sp., *Porphyromonas* sp. oral taxon 930, *Bacillus subtilis*, *Lactobacillus* sp., *Haemophilus* sp., *Kingella* sp., *Capnocytophaga granulosa*, *Campylobacter showae*, *Alloprevotella* sp., *Selenomonas noxia*, *Cardiobacterium* sp., *Corynebacterium matruchotii*, *Alloprevotella* sp. oral taxon 473/taxon 912, *Prevotella maculosa*, *Treponema* sp., *Actinomyces* sp. oral taxon 525, *Capnocytophaga* sp. oral taxon 332, *Neisseria elongata*, *Ralstonia* sp., *Kingella denitrificans*, *Neisseria subflava*, *Treponema maltophilum*, *Fusobacterium* sp., *Aggregatibacter* sp., *Aggregatibacter* sp. oral taxon 458, *Campylobacter concisus*, *Cardiobacterium valvarum*, *Eikenella corrodens*, *Campylobacter rectus*, *Actinomyces* sp. oral taxon 897/taxon 408, *Cardiobacterium hominis*, *Prevotella* sp. oral taxon 317, *Campylobacter* sp., *Bacillus* sp., *Campylobacter gracilis,* and *Bergeyella* sp. oral taxon 900 was discovered when compared to others.

The data for the SE.S group showed a higher proportion of *Prevotella histicola*, *Prevotella* sp. oral taxon 305, *Atopobium parvulum*, *Leptotrichia* sp. oral taxon 215, *Actinomyces* sp. oral taxon 172, *Mogibacterium* sp., *Granulicatella* sp., *Atopobium* sp., *Peptostreptococcus stomatis*, *Streptococcus vestibularis*, *Actinomyces lingnae* NVP, *Prevotella melaninogenica*, *Rothia mucilaginosa*, *Rothia* sp., *Peptostreptococcus* sp., and *Granulicatella adiacens* than others. As for the samples from the SE.F group, a higher proportion of *Tannerella* sp. oral taxon 286, *Abiotrophia* sp., *Ottowia* sp. oral taxon 894, *Porphyromonas* sp., *Lautropia* sp., *Fusobacteria* sp., *Leptospira* sp., *Lautropia mirabilis*, *Abiotrophia defectiva*, *Gemella morbillorum*, and *Tannerella* sp. was found. In the carious dental plaque samples (SE.D group), it showed that *Pseudomonas* sp., *Neisseria flava*, *S. mutans*, *Actinomyces* sp. oral taxon 448/taxon 170/taxon 169, *Prevotella* sp. oral taxon 472, *Kingella oralis*, and *Haemophilus parainfluenzae* accounted for a higher proportion.

#### Fungi

As shown in Fig. 4B, the data showed that samples from the CF.S group accounted for a higher proportion of *Hymenochaete* sp., *Candida pseudoglaebosa*, *Candida santamariae*, and *Hymenochaete* sp. than others. *Malasseziales* sp. were detected with a higher proportion in the healthy dental plaque samples (CF.F group).

The data for the SE.S group showed a higher proportion of *Nigrospora* sp., *Candida* sp., *Phoma* sp., *Lodderomyces elongisporus*, *Nigrospora oryzae*, *Phoma leveillei*, *Sterigmatomyces* sp., *Wickerhamomyces anomalus*, *Ascomycota* sp., *Sterigmatomyces halophilus*, *Dipodascus australiensis*, *Wallemia* sp., *Lodderomyces* sp., and *Candida tropicalis* than others. A higher proportion of *Aspergillus restrictus*, *Penicillium* sp., *Aspergillus* sp., *Ascotricha guamensis*, *Aspergillus gracilis*, *Apiotrichum xylopini*, *Aspergillus conicus,* and *Aspergillus penicillioides* were detected in the SE.F samples when compared to the others. The fungi that were significantly more abundant in the plaque of the carious tooth surface (SE.D group) were *C. albicans*, *Pleosporales* sp., *Pichia kluyveri,* and *Wallemia canadensis*.

### Environmental factor association analysis of fungal and bacterial communities in the SE and CF groups

The values of the relevant environmental factors for the SE and CF groups are shown in Table 4. The *t*-test analysis showed that there was a significant difference (*P* < 0.05) in salivary pH and fluoride concentration between the two groups. The mean values of salivary pH and fluoride concentration in the CF group were significantly higher than those in the SE group.

**TABLE 4:** Environmental factor values for the CF and SE groups^*a*^

Sample ID	Group	Iron concentration (µg/L)	Fluoride concentration^**^ (mg/L)	Salivary pH^*^	Buffering capacity (ppm)
CF.1	CF	2.45	0.060	6.5	250
CF.2	CF	25.26	0.198	6.5	250
CF.3	CF	10.99	0.090	5.5	250
CF.4	CF	13.88	0.144	6.0	250
CF.5	CF	10.49	0.102	7.0	250
CF.6	CF	21.87	0.138	6.0	250
CF.7	CF	15.90	0.072	6.5	500
CF.8	CF	44.96	0.144	7.0	500
CF.9	CF	41.60	0.096	6.5	250
CF.10	CF	3.98	0.210	6.0	250
CF.11	CF	15.84	0.096	6.5	250
CF.12	CF	9.44	0.150	6.5	250
CF.13	CF	10.56	0.060	5.5	250
CF.14	CF	35.24	0.096	6.5	500
CF.15	CF	15.36	0.132	6.5	500
CF.16	CF	6.25	0.096	7.0	500
CF.17	CF	14.55	0.162	6.0	250
CF.18	CF	11.29	0.156	6.0	250
CF.19	CF	19.47	0.174	7.0	250
CF.20	CF	9.67	0.186	6.5	250
CF.21	CF	25.89	0.174	6.0	250
CF.22	CF	6.85	0.228	7.0	500
CF.23	CF	11.40	0.096	7.0	250
CF.24	CF	25.28	0.168	6.0	250
CF.25	CF	12.49	0.144	6.0	250
CF.26	CF	9.74	0.108	6.5	250
CF.27	CF	10.15	0.186	6.5	500
CF.28	CF	18.27	0.174	7.0	500
CF.29	CF	10.78	0.108	7.0	500
CF.30	CF	18.66	0.108	6.5	250
CF.31	CF	7.81	0.120	8.0	500
CF.32	CF	9.40	0.084	7.0	500
CF.33	CF	5.22	0.150	6.5	250
SE.1	SE	21.41	0.078	6.5	500
SE.2	SE	32.29	0.072	6.5	250
SE.3	SE	9.66	0.090	8.0	500
SE.4	SE	15.44	0.090	7.0	500
SE.5	SE	5.17	0.096	6.5	250
SE.6	SE	34.15	0.084	5.5	<20
SE.7	SE	5.78	0.096	5.5	<20
SE.8	SE	27.06	0.150	7.0	250
SE.9	SE	1.95	0.102	6.5	500
SE.10	SE	11.79	0.132	5.5	<20
SE.11	SE	0.65	0.102	5.5	500
SE.12	SE	9.33	0.132	5.5	250
SE.13	SE	5.12	0.072	5.5	500
SE.14	SE	27.37	0.066	6.5	250
SE.15	SE	11.76	0.072	6.0	250
SE.16	SE	13.62	0.108	6.5	500
SE.17	SE	18.02	0.108	6.5	500
SE.18	SE	57.13	0.084	5.5	250
SE.19	SE	8.91	0.132	6.0	250
SE.20	SE	22.79	0.078	6.0	500
SE.21	SE	15.41	0.150	5.5	250
SE.22	SE	11.99	0.084	6.0	250
SE.23	SE	6.46	0.096	5.5	<20
SE.24	SE	14.24	0.084	5.5	250
SE.25	SE	29.74	0.096	6.0	250
SE.26	SE	17.98	0.096	8.0	500
SE.27	SE	8.50	0.114	5.5	250
SE.28	SE	14.03	0.126	5.5	250

^*a*^ *t*-test; *P < 0.05, **P < 0.01.

#### Correlation analysis of iron and fluorine levels with bacterial and fungal communities

**Fig 5:**
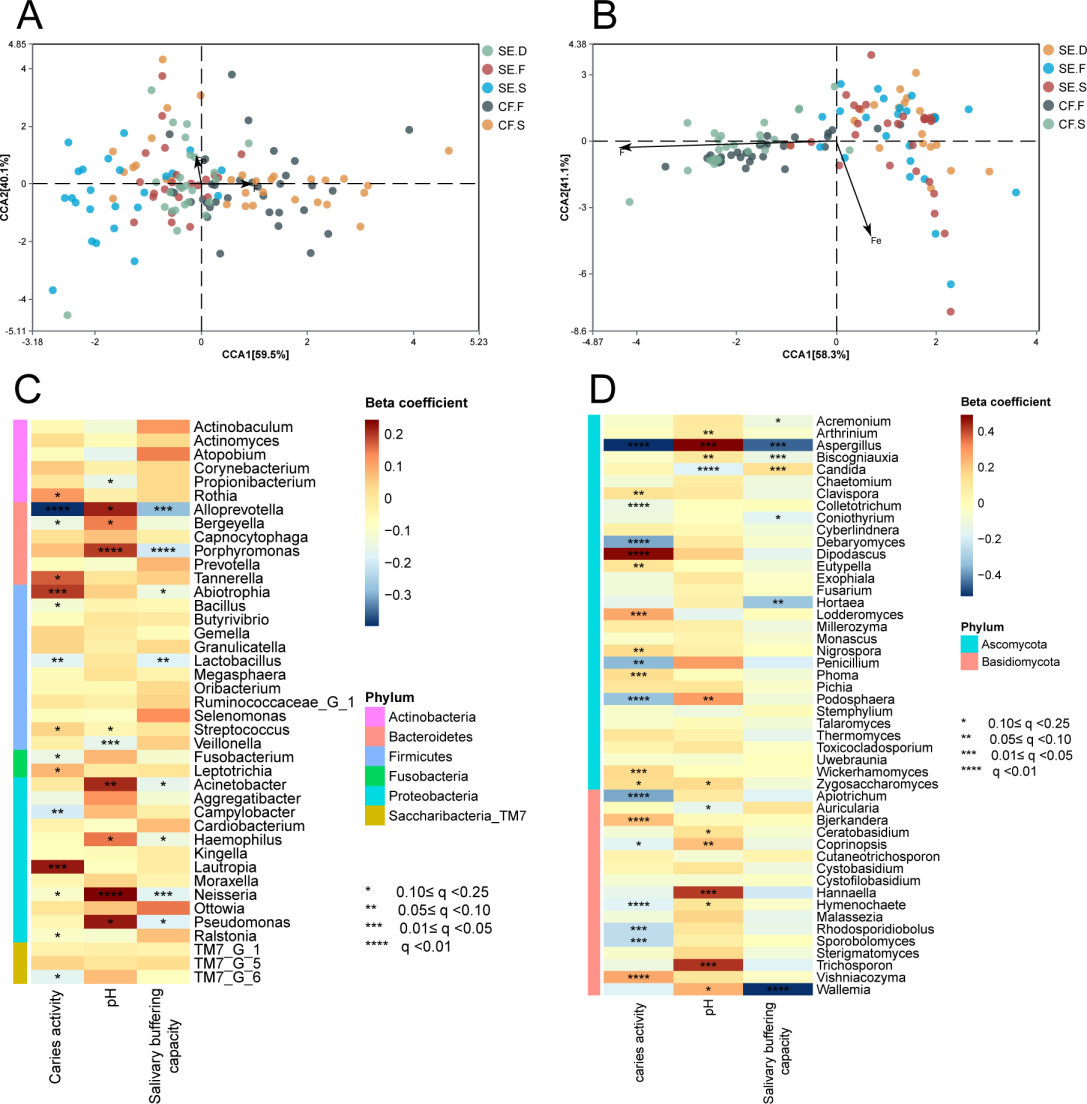
Microenvironmental factors analysis. RDA of the microbial communities and iron and fluorine concentrations at each spatial site in the SE and CF groups. (**A**) Bacteria and (**B**) fungi. The arrows in the figure represent iron and fluorine, and the different dots represent the samples. The correlation is represented by the angle between the arrows. The length of the arrows represents the magnitude of the correlation, with longer arrows indicating a greater effect on the microorganisms. MaAsLin analysis of salivary pH and buffering capacity of bacterial communities (**C**) and fungal communities (**D**).

The results of RDA (Fig. 5A and B) showed that Fe and F exhibited a negative correlation. However, there was no significant correlation between iron and fluorine levels or between the distributions of bacterial and fungal communities in the SE group and the CF group (*P* > 0.05).

#### Correlation analysis of salivary pH and buffering capacity with bacterial communities

The results of the MaAsLin analysis are shown in Fig. 5C: *Alloprevotella* sp., *Bergeyella* sp., *Porphyromonas* sp., *Acinetobacter* sp., *Neisseria* sp., and *Pseudomonas* sp. are positively correlated with pH, while *Veillonella* sp. and *Propionibacterium* sp. are negatively correlated. For example, *Neisseria* sp. are positively correlated with pH, which means that when the pH value increases, *Neisseria* sp. tends to exhibit a higher abundance (q < 0.01). *Alloprevotella* sp., *Porphyromonas* sp., and *Lactobacillus* sp. were negatively correlated with salivary buffering capacity (*P* < 0.05).

#### Correlation analysis of salivary pH and buffering capacity with fungal communities

The results of the MaAsLin analysis are shown in Fig. 5D: *Aspergillus* sp., *Biscogniauxia* sp., *Podosphaera* sp., *Hannaella* sp., and *Trichosporon* sp. were positively correlated with pH, while *Candida* sp. was negatively correlated with pH and positively correlated with salivary buffering capacity. The abundance of *Aspergillus* sp., *Biscogniauxia* sp., *Coniothyrium* sp., *Hortaea* sp., and *Wallemia* sp. were significantly negatively correlated with the salivary buffering capacity (*P* < 0.05).

### Network analysis and correlation analysis of bacteria and fungi

#### Network analysis within bacteria or fungi

**Fig 6:**
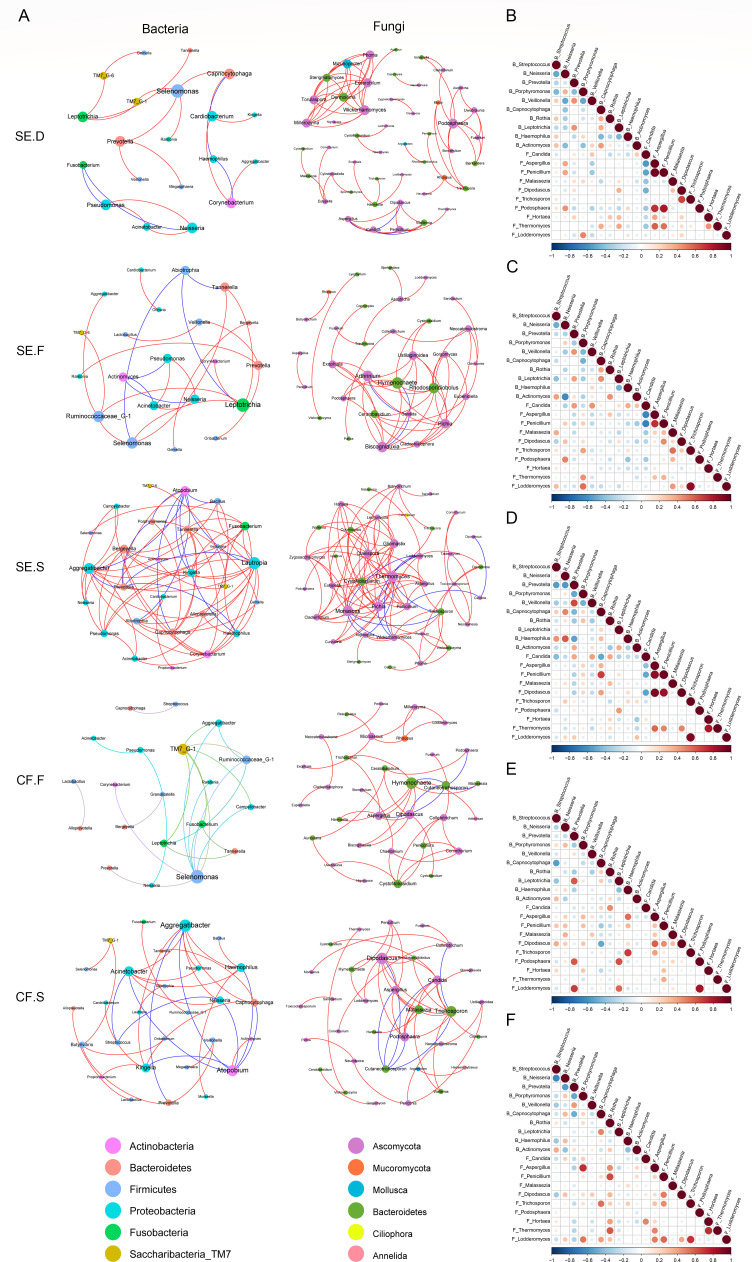
Network analysis and correlation analysis of bacteria and fungi. Network analysis of bacteria and fungi within each site of the SE group and CF group (**A**). A line between two species indicates a significant correlation (Spearman’s rank correlation *ρ >* 0.6, *P <* 0.01). The greater the number of lines between nodes and the larger the radius of the nodes are, the greater the correlation. Red represents a positive correlation between species, and blue represents a negative correlation between species. Correlation analysis of bacteria and fungi within each site of the SE group and CF group (B, SE.D; C, SE.F; D, SE.S; E, CF.F; F, CF.S). "B_ stands for bacteria" and "F_ stands for fungi." Red and blue represent positive and negative correlations, respectively. The color shade and size of the circles are proportional to the correlation coefficient, and white represents no correlation.

The analysis revealed interactions between 168 bacterial (SE.D:21, SE.F:23, SE.S:68, CF.F:22, CF.S:34) and 313 fungal genera (SE.D:71, SE.F:50, SE.S:104, CF.F:42, CF.S:46). Figure 6A shows that the number of nodes and edges between bacterial communities in plaque samples were less than those in saliva samples in both the SE and CF groups. In the SE and CF groups, most bacteria-bacteria and fungi-fungi genera were positively correlated. Compared with fungi, there were more species that were negatively correlated with the bacterial communities of caries plaque. In Fig. 6, larger fungal co-occurrence networks also had a higher number of nodes and a greater number of paths between nodes than bacterial networks, suggesting that interactions between fungal networks are more complex than bacterial networks.

#### Correlation analysis of bacteria and fungi

The correlation analysis provides insights into the ecological interactions and potential competition or cooperation between bacterial and fungal species at different spatial locations within the SE and CF groups. Figure 6B through F show the correlations among the five spatial sites. The results revealed significant differences in the correlation patterns between bacterial and fungal species within each spatial location. For instance, in the SE.D group, *Streptococcus* sp. exhibited negative correlations with *Neisseria* sp., *Prevotella* sp., *Porphyromonas* sp., *Malasseziales* sp., *Candida* sp., *and Hortaea* sp.*,* but showed positive correlations with *Veillonella* sp., *Rothia* sp., *Actinomyces* sp., *Aspergillus* sp., *Penicillium* sp., *and Trichosporon* sp. However, in the SE.F and SE.S groups, *Streptococcus* sp. positively correlated with *Malassezia* sp. and *Hortaea* sp.

## DISCUSSION

Our research revealed a significant difference in the bacterial and fungal communities in the SE group, which included samples of healthy dental plaque, dental caries plaque, and saliva, compared to those in the CF group, which consisted of healthy dental plaque and saliva samples. Interestingly, the SE group showed distinct bacterial and fungal community structures in healthy and carious dental plaque compared to those in saliva, indicating that the occurrence of carious lesions causes changes in the microbial community configurations at each respective site. However, we found that within the CF group, no significant difference was observed in the fungal compositions of healthy dental plaque and saliva samples (*P* > 0.05).

*C. albicans* exhibited the highest fungal abundance in the SE group, with percentages reaching 59.2% in SE.S, 71.8% in SE.F, and 77.1% in SE.D. This finding aligns with previous studies (3) that highlighted *C. albicans* as one of the most frequently cultured oral fungi, which has unique potential for causing dental caries. *C. albicans* can invade dentin tubules, leading to the demineralization and dissolution of teeth (48, 49). Recent researches on *Candida* has demonstrated its status as an acid-producing and acid-tolerant fungus, capable of independently causing caries (50–52). A meta-analysis (53) based on nine cross-sectional epidemiological studies revealed that children who tested positive for *C. albicans* had a fivefold higher risk of early childhood caries (ECC) than those who tested negative. Furthermore, *C. albicans* can collaborate with other bacteria and fungi, enhancing biofilm formation, aggravating periapical inflammation when coexisting with *Enterococcus faecalis* (12–14), and amplifying its cariogenic capability when present alongside *S. mutans* (15, 16, 54–56).

In the CF group, Tremellomycetes sp. was found to be the most abundant fungus, accounting for 86.2% in CF.S and 72.0% in CF.S. Notably, this fungus has not been previously reported in any studies. Therefore, more research is needed to determine whether this species is transient or permanent within the oral cavity. Furthermore, *Malasseziales* sp. was significantly higher in healthy dental plaque samples (*P* < 0.05). Previous studies have indicated that *Malasseziales* sp. is an important oral symbiotic fungus present in both saliva and healthy dental plaque (57–60). Moreover, studies have shown that a diet low in carbohydrates and high in lipids, along with good oral hygiene practices, favors the growth of *Malassezia* (61). These findings suggest that it may play a significant role in maintaining oral microecological health.

In terms of bacterial composition, the abundance of *S. mutans* in the saliva of the SE group was significantly higher than that in the CF group (*P* < 0.05). This finding aligns with a previous systematic review that highlighted *S. mutans* as more abundant in caries-affected individuals (62). Microscopic *in situ* evaluations of intact clinical biofilm samples from caries-affected individuals have suggested a pivotal role for *Candida* in the biofilm development of cariogenic bacteria (54). These studies elucidated the interactions between fungi and bacteria, with imaging data depicting bacteria from *S. mutans* clustering around *C. albicans*, thereby forming distinctive fungal-bacterial aggregates that resemble corncobs in morphology. This co-aggregation significantly enhances the biofilm-forming capacity of *S. mutans* and *C. albicans* in both *in vitro* and *in vivo* settings (63). The presence of *C. albicans* increased the production of exopolysaccharides, which allowed the co-species biofilms to accumulate more biomass and contain more active *S. mutans* cells than single-species biofilms (64). Co-infection synergistically enhances the pathogenicity of the biofilm, leading to aggressive disease episodes and extensive carious lesions.

Furthermore, notable increases were observed in bacteria belonging to *Granulicatella* and *Actinomyces* (oral taxon 448, taxon 170, and taxon 169) within the SE group. *Granulicatella* is a facultative anaerobic Gram-positive cocci bacteria that can ferment glucose to produce lactic acid (65). Previous studies have established a link between *Granulicatella* and caries, particularly S-ECC (66, 67). Nevertheless, the intricate mechanism underlying caries formation involving *Granulicatella* remains elusive. On the other hand, *Actinomyces* is recognized as a significant pathogenic microorganism that contributes to root surface caries. *Actinomyces viscosus* has a swift ability to adhere to the root surface and bind to the collagen fibers of dentin and cementum, playing a pivotal role in regulating mineral loss on the tooth surface (68–70). Based on the above research findings, it is highly likely that these two bacteria are involved in the pathogenic process of dental caries. This hypothesis urgently requires further rigorous verification through *in vitro* experiments.

The correlation analysis of bacteria and fungi revealed complex interactions between these two microbial groups in the SE and CF groups. Notably, fungal communities exhibited a more complex network of interactions than did bacterial communities. In line with previous research findings (1, 2, 71, 72), our results suggest that bacteria and fungi in the oral microenvironment engage in intricate interactions that contribute to the stability of the oral ecosystem or participate in pathogenic processes. Specifically, we observed that pairs of microorganisms that are positively or negatively correlated in the healthy state may show the opposite correlation in the caries state. For instance, *Streptococcus* was negatively correlated with *Hortaea* in carious plaque samples but positively correlated in healthy plaque and saliva samples. This intriguing discovery requires further identification and verification to elucidate its underlying mechanisms.

The results of this study demonstrated that the salivary pH value in the CF group was significantly higher than that in the SE group (*P* < 0.05). This finding is in accordance with the conclusions of previous studies that have shown a negative correlation between salivary pH and the decayed, missing, filled teeth (DMFT) index (73). The dental plaque pH and salivary pH are important environmental factors that affect the microenvironment of dental biofilms (28). The development of caries is associated with a decrease in pH in the microenvironment and an increase in acid-producing and acid-resistant bacteria (74, 75). Furthermore, we found a negative correlation between the pH value (5.5–8) and the abundance of *Veillonella, Propionibacterium*, and *Candida*.

*Veillonella* is among the earliest colonizers of dental plaque and utilizes lactic acid produced by other bacteria, such as *S. mutans*, as a carbon source and produces substances such as acetic acid (76, 77). Studies have demonstrated that genes involved in lactic acid and succinic acid catabolism, as well as histidine biosynthesis, are upregulated in caries tissue samples compared to healthy saliva samples. This upregulation may contribute to the survival of *Veillonella* in an acidic environment (78). However, a previous cross-sectional study about preschoolers shows that *Veillonella* showed higher abundance in the caries-free group than the rampant caries group and positively correlated with the salivary pH levels (79). Longitudinal studies are required to confirm the relationship between *Veillonella* and dental caries.

*Propionibacterium* has been reported to be a dominant factor in dentin caries (80, 81). Studies have highlighted the significant role of *Propionibacterium* in caries progression (82). However, another study using similar molecular techniques to determine the microbial diversity in adults with advanced caries found that the abundance of *Propionibacterium* species was not commonly detected (83). Therefore, the relationship between *Propionibacterium* and caries requires further investigation.

Furthermore, our study revealed a negative correlation between iron and fluoride concentrations. However, there was no significant difference in the distribution of oral bacterial and fungal communities associated with iron and fluoride concentrations in either group. Previous studies have identified iron as an essential nutrient for bacteria and fungi (32, 33). Animal experiments have suggested that iron may inhibit the progression of dental caries (36, 37). Clinical studies have demonstrated an association between low iron concentration and high DMFT (73). However, the underlying mechanisms have not been fully elucidated.

Fluoride is a highly effective element for preventing ECC (39). It works by inhibiting enamel demineralization and promoting remineralization, which helps prevent cavities (40, 41). The antimicrobial and anticariogenic effects of fluoride are mainly due to its ability to reduce the acid tolerance of glycolysis by cariogenic bacteria in plaque (84). Nevertheless, similar to our study, fluoride varnish did not have a significant effect on oral organisms in preschool children (85). A possible explanation is that while many organisms are sensitive to fluoride in free or simple biofilm models *in vitro*, their responses may vary within complex oral biofilm communities (84, 86).

In our study, a statistically higher proportion of children in the SE group were born by caesarean section and breastfed in the first 6 months of life. These findings are consistent with the conclusions of some previous clinical studies (87, 88). Previous evidence suggested that the mode of delivery could have an impact on the establishment of the infant oral microbiome, which in turn may affect the risk of dental caries (87, 88). Infants born vaginally gain an advantage as they are exposed to the maternal oral and gastrointestinal microbiota during the birthing process. This exposure might contribute to the development of a more beneficial oral microbiome and a reduction in the abundance of caries-causing pathogens. Conversely, infants delivered by cesarean section are deprived of this natural transmission pathway and instead acquire oral bacteria from alternative environmental sources (89). Alternatively, previous meta-analysis studies (89, 90) suggest that no significant association was found between mode of delivery and ECC. Thus, to more definitively determine the relationship between delivery and ECC, high-quality research evidence is essential.

The relationship between the duration of breastfeeding and the risk of dental caries is rather complex. While short-term breastfeeding (less than 6 months) is generally considered safe, long-term breastfeeding (over 12 months) may increase the risk of dental caries (91, 92). This does not conflict with the finding of our study. The limitation is that we only collected data on the feeding patterns of children within the first 6 months of age. If the data collection period were extended to 12 months or longer, we might obtain more information on the relationship between breastfeeding duration and caries.

Whether the caries lesion itself changes the microbial community or whether the microbial community on the tooth causes the lesion is a very important scientific question. Furthermore, when the bacterial-fungal interaction is focused on specific strains or genera, how exactly does their relationship change, and by what mechanism? What role does this change play in the development of caries? Based on the cross-sectional studies we have conducted so far, we are able to observe phenomenological changes between caries status and microbial communities, but specific causal analyses do need to be confirmed by further prospective longitudinal studies. These studies will help us understand the chronological and causal relationships between changes in microbial communities and the development of caries, thus providing a more in-depth scientific basis for caries prevention and treatment.

### Conclusions

To date, limited research has been conducted on the relationships between salivary pH, iron, fluorine, and oral microbial communities. More studies are necessary to investigate the connections between these factors and oral bacterial and fungal communities.

Our research discovered differences in the structures of fungal and bacterial communities present in the oral saliva and dental plaque of children suffering from S-ECC. The distribution of these communities varied depending on the state of caries. We identified complex interactions among fungi and bacteria present in both healthy and carious states. S-ECC children had significantly higher levels of bacteria such as *S. mutans*, *Granulicatella*, and *Actinomyces*, as well as fungi such as *C. albicans* than caries-free children. We found no significant correlation between the concentrations of iron and fluoride and the oral bacterial and fungal communities (*P* > 0.05).

Bacteria such as *Veillonella, Propionibacterium,* and *Leptotrichia,* as well as fungi including *Nigrospora, Candida*, and *Bjerkandera*, were found to be associated with a decrease in salivary pH value.

## Data Availability

The data sets generated during the current study are available in the SRA repository with the accession numbers PRJNA1080488 and PRJNA848441.

## SUPPLEMENTAL MATERIAL

Supplemental material spectrum.02518-24-s0001.docx Oral Health Survey Questionnaire.

ASM does not own the copyrights to Supplemental Material that may be linked to, or accessed through, an article. The authors have granted ASM a non-exclusive, world-wide license to publish the Supplemental Material files. Please contact the corresponding author directly for reuse.
