# Supplementary material for: Inter-kingdom interactions and environmental influences on the oral microbiome in severe early childhood caries
Source: Microbiol Spectr. 2025 Apr 15;13(6):e02518-24. doi: 10.1128/spectrum.02518-24 (PMC12131756; doi:10.1128/spectrum.02518-24)
Supplement: Supplemental material — Oral Health Survey Questionnaire. [file spectrum.02518-24-s0001.docx]

Oral Health Survey Questionnaire

Please complete this form in full.

| Child's Name: __________ | Gender: __________ 1=Male 2=Female |
| --- | --- |
| Date of Birth: __________ | Contact Phone Number: __________ |

(Please tick the box in front of the chosen answer: **🗹** )

First, let's understand some information about your child:

1. Current weight of your child: ________ kilograms, height: ________ centimeters;
2. Has your child used antibiotics in the last 6 months?

⬜ Yes ⬜ No

1. What was your child's birth weight?

⬜ Below 1.5 kilograms

⬜ 1.5 to 2.5 kilograms

⬜ Above 2.5 kilograms

1. How far along was your pregnancy?

⬜ 28-36 weeks

⬜ More than 37 weeks

1. What was the mode of delivery of your child?

⬜ Natural delivery

⬜ Caesarean section

1. What was your child's feeding method within 6 months after birth?

⬜ Breastfeeding

⬜ Mixed feeding

⬜ Formula feeding

1. At what age did your child stop the habit of night feeding?

⬜ Before 1 year

⬜ 1-2 years

⬜ After 2 years

1. Do you or any family members have the habit of chewing food and then feeding it to the child?

⬜ Yes

⬜ No

1. How often does your child brush their teeth?

⬜ Less than 1 time/day

⬜ 1 time/day

⬜ 2 or more times/day

1. Do you use fluoride toothpaste to clean your child's teeth?

⬜ Yes

⬜ No

⬜ Not sure

1. Has your child ever visited the dentist?

⬜ Yes

⬜ No

⬜ Not sure

(If the answer is "Yes", please specify the reason for the dental visit: ________)

1. How often does your child consume the following foods?

|  | Less than once a week | 1-3 times a week | 4-6 times a week | More than once a day |
| --- | --- | --- | --- | --- |
| Sweets (such as chocolate, candy, cake, etc.) |  |  |  |  |
| carbonated soft drinks  (such as cola, sprite, etc.) |  |  |  |  |
| Sweetened drinks (such as milk tea, fruit juice, etc.) |  |  |  |  |

1. How do you rate your child's oral condition? Please provide a score.

Oral health level: Very poor Average Very good

| 1  ⬜ | 2  ⬜ | 3  ⬜ | 4  ⬜ | 5  ⬜ |
| --- | --- | --- | --- | --- |

Score:

Finally, some questions about your family's basic situation:

1. Are you the child's:

⬜ Mother

⬜ Father

⬜ Other relative

1. Your family's average monthly income is approximately:

⬜ Less than 1000 yuan

⬜ 1000-4999 yuan

⬜ 5000 yuan or more

Thank you very much for your assistance! We wish your family health and happiness!
